# Immunosensor for Assessing the Welfare of Trainee Guide Dogs

**DOI:** 10.3390/bios11090327

**Published:** 2021-09-09

**Authors:** Hannah Perkins, Michelle Higgins, Marinara Marcato, Paul Galvin, Sofia Rodrigues Teixeira

**Affiliations:** 1 Tyndall National Institute, University College Cork, T12 R5CP Cork, Ireland; hannahcp811@gmail.com (H.P.); michelle17higgins@gmail.com (M.H.); marinara.marcato@tyndall.ie (M.M.); paul.galvin@tyndall.ie (P.G.); 2 School of Chemistry, University College Cork, T12 YN60 Cork, Ireland

**Keywords:** cortisol, dogs, stress, sensor, graphene, impedance

## Abstract

Cortisol is a well established biomarker hormone that regulates many processes in the body and is widely referred to as the stress hormone. Cortisol can be used as a stress marker to allow for detection of stress levels in dogs during the training process. This test will indicate if they will handle the stress under the training or if they might be more suitable as an assistant or companion dog. An immunosensor for detection of cortisol was developed using electrochemical impedance spectroscopy (EIS). The sensor was characterized using chemical and topographical techniques. The sensor was calibrated and its sensitivity determined using a cortisol concentration range of 0.0005 to 50 μg/mL. The theoretical limit of detection was found to be 3.57 fg/mL. When the immunosensor was tested on canine saliva samples, cortisol was detected and measured within the relevant physiological ranges in dogs.

## 1. Introduction

Guide dogs have an important role as they are the eyes of their visually impaired companion. However, not all dogs are capable or suitable to function as a guide dog. A guide dog’s ability to guide an individual can be affected by external factors in their surroundings, such as loud noises, social interactions and environmental factors [1]. Certain dogs can deal with stressful situations better than others, and thus it is important for both organizations and trainers to better understand the level of stress that a guide dog is experiencing. Stress results in a higher level of cortisol present in their saliva [2]. Several factors can affect canine cortisol levels including both physical and cognitive activity. Cortisol levels in dogs may also indicate if a dog is suffering from an illness. Hypercortisolism and hypocortisolism are medical conditions resulting from excess and deficient cortisol levels in dogs, respectively [3]. Cortisol levels in working dogs have been found to increase as a result of their working activities [1], in addition to health conditions such as those noted above. Hence, this parameter could be useful towards informing the selection of dogs which undergo the expensive and resource consuming guide-dog training process, in order to optimize the success rate for selecting dogs appropriately. Biosensors have been tested with saliva and other body fluids such as blood, interstitial fluid and urine, all of which will give the amount of cortisol present [4]. The stress response of a number of animals has been assessed through saliva analysis, owing to the link between saliva and plasma cortisol concentrations. Moreover, saliva sampling offers a non-invasive means of assessing animal behavior [5,6,7]. There are a number of methods and indications that can be used to assess a canine’s stress levels [8]; however, salivary cortisol is one of the more commonly used techniques to measure the stress levels in animals. 

Several studies using human salivary have been developed. Kämäräinen et al. [9] developed an SPE coated with a cortisol-alkaline phosphatase conjugate and using square wave voltammetry; the sensor showed a working concentration range of 0.2 to 44.6 ng/mL. The results were highly reproducible within the desired physiological range for human salivary cortisol and showed a 0.90 correlation in results when compared with ultra-high pressure liquid chromatography tandem mass spectrometry [9]. Recent research states the potential use of dual electrochemical sensor systems (for cortisol and lactate) using an electro-reduced graphene oxide screen-printed electrode, also showing high sensitivity and specificity [10].

Enzyme-linked immunosorbent assay (ELISA) is the most used technique for detection of cortisol levels because of the sensitivity and versatility to evaluate protein concentrations [11]. The development of a reliable, non-invasive point of care is needed to help animals with stress-related problems. These types of point of care can deliver quick real-time cortisol values [12].

Saliva has been used in a number of studies to evaluate dog and animal welfare [5,13,14,15,16]. Cobb et al., documented a cortisol concentration range of 0 to 337.9 ng/mL, with a mean of 4.5 ng/mL and a median of 1.5 ng/mL on studying meta-analysis of salivary cortisol levels [1]. Plasma cortisol and salivary cortisol levels in dogs are very closely related and it has been demonstrated that if the collection time takes less than 4 min, there will be no handling effect on the cortisol concentration [17]. Cobb et al. stated that no significant variations were evident in salivary cortisol concentrations of different dogs based on body weight or coat color or type (i.e., pet, assistance or therapy dog, military etc.), but found that unneutered females generally have higher cortisol concentrations than neutered females, males and unneutered males [1]. In an investigation by Batt et al. [18] cortisol concentrations were measured using an enzyme immunoassay from the saliva samples of potential guide dogs at 6 months of age and 14 months of age (before training commenced). Once a dog was determined to be suitable to work as a guide dog, cortisol concentrations were measured again using the same procedure. The study noted that at 6 months of age, dogs with higher cortisol levels were found to be stressed when in an unfamiliar environment. Furthermore, once training was complete the cortisol levels were found to be significantly higher in comparison to measurements obtained prior to training. However, it was unclear what exactly this elevation reflected. It may have been a result of maturation or the effect of prolonged kenneling during the training period.

Another study examined cortisol levels in animal-assisted activity dogs to monitor the welfare of these dogs [19]. Cortisol levels were found to be significantly raised when the dogs were exposed to a novel setting (3.97 ng/mL) compared to a home setting (2.13 ng/mL) or after activity without interaction with a stranger (2.57 ng/mL). The methodology applied in that research is not dissimilar to this study-cortisol samples were collected before, during and after 60-min training sessions (across three different settings). For this study, the samples were collected from the trainee guide dogs also before and after a behaviour assessment session. Immunosensors have a number of advantages over conventional analytical methods, including increased detection ranges, higher sensitivities in addition to rapid selective detection. Hence, they are an attractive and appropriate technique for evaluating stress levels [20,21].

Electrochemical sensors have been reported in the literature for the detection of cortisol, but only few of these are related to dogs [14]. Both antibodies and aptamers can be used to assemble cortisol sensors as can be seen in Table 1 [9,22,23,24,25,26,27,28,29,30,31,32,33,34,35,36,37,38,39,40,41,42,43,44].

In this work, we describe a successful label-free method for ultrasensitive cortisol detection using an electrochemical impedance immunosensor in canine saliva obtained from Irish Guide Dogs for the Blind (IGDB). Here, we show that polyaniline (PANI) electrodeposited on graphene can be used for EIS cortisol analysis in both buffer and dog saliva samples. A linear response to cortisol concentrations between 0.0005 and 50 μg/mL, and an LOD of 3.57 fg/mL was achieved.

## 2. Experimental

### 2.1. Materials and Equipment

*N*-Hydroxysuccinimide, bovine serum albumin (BSA), Phosphate Buffered Saline (PBS), aniline solution, sulphuric acid (H_2_SO_4_), cortisol antigen, Potassium hexacyanoferrate III (K_3_[Fe(CN)_6_]), potassium hexacyanoferrate II (K_4_[Fe(CN)_6_]) trihydrate and N-(3-Dimethylaminopropyl)-N’-ethylcarbodiimide hydrochloride were purchased from Sigma Aldrich (Gillingham, UK); monoclonal anti-cortisol antibody (Ab) was purchased from Santa Cruz Biotechnology (Dallas, TX, USA). Aniline solution (0.1 M) was prepared in H_2_SO_4_ (1 M). PBS solutions were prepared by dissolving 1 PBS tablet in 200 mL of de-ionized water. Deionized water (18.2 MΩ/cm) was used in all the experimental work.

Electrochemical impedance spectroscopy (EIS) and Cyclic voltammetry (CV) were done with a potentiostat, Metrohm Autolab (Utrecht, The Netherlands), MAC90389, in connection to a laptop and measurements were carried out using Nova software. Graphene-SPEs were obtained from DropSens (DRP-110GPH). Raman measurements were done with a Renishaw InVia system with a wavelength of 514 nm and power of 7 mW at 100%, using an objective magnification of 50. SEM were completed with an FEI Quanta 650 SEM (ThermoFischer Scientific, Waltham, MA, USA).

### 2.2. Fabrication of the Immunosensor

**Figure 1:**
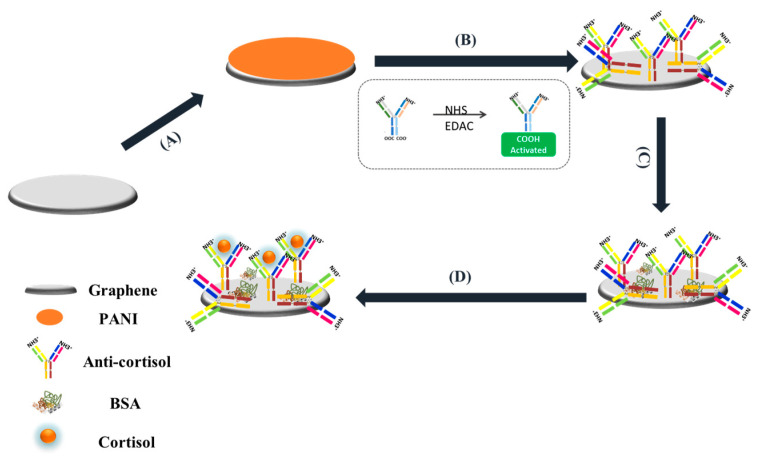
Schematic of the immunosensor assembly.

PANI deposition was performed as described in our previous work [45]. Briefly, PANI was obtained by electropolymerization of 0.1 M aniline, with a potential sweep between −0.1 and +1.2 V, 50 mV/s for 20 cycles. The working electrode (WE) was rinsed with PBS prior to adding 8 μL of anti-cortisol on the surface (incubating a 100 µg/mL antibody solution in 25 mmol/L EDAC and 50 mmol/L of NHS). The immobilization of antibodies using the NHS/EDAC method does not ensure that the antibody is in the correct orientation after immobilisation—some of binding sites may not be available for cortisol—but helps to improve the binding. In addition, the cortisol should bind to the binding sites of antibodies at their Fab parts, not between them. The sensor was then left at room temperature for 2 h. BSA (0.5 mg/mL) was also added to the modified WE for 30 min at room temperature to prevent unspecific surface binding (Figure 1). The modified surface was stored at 4 °C until testing commenced.

### 2.3. Characterization and Measurements

The cortisol levels in the dogs’ saliva samples and the surface functionalization process were evaluated and performed by EIS and CV. CV measurements were performed in 5.0 mmol/L of [Fe(CN)_6_]^3−^/[Fe(CN)_6_]^4−^ at 50 mV/s from −0.7 V to +0.7 V. EIS were tested using an amplitude of 100 mV at a potential of +0.10 V, and a frequency equivalent to 50 Hz, with a frequency range of 1000 Hz–0.05 Hz. The impedance data were fitted to a R(C[R(W)]) Randles equivalent circuit using the Nova Software.

The WE was covered with 8 μL of sample solution and incubated at room temperature for 15 min. Cortisol was also tested in canine saliva samples.

### 2.4. Canine Saliva Handling Process

Sample were collected from the dogs at Irish Guide Dogs for the Blind, then centrifuged at 2000 rpm for 10 min. From this, the supernatant solution was collected and 8μL was placed onto the WE sensor. Samples were kept at −20 °C until use.

## 3. Results and Discussion

### 3.1. Characterization of the Immunosensor Surface Chemistry

**Figure 2:**
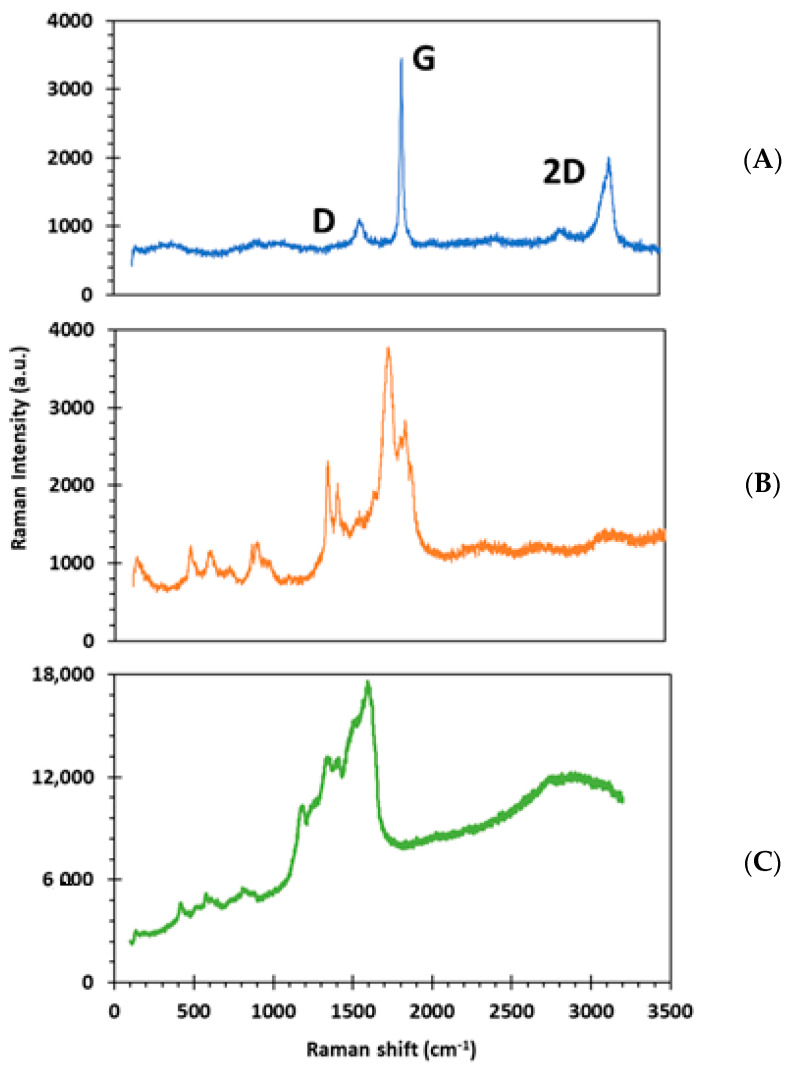
Surface chemistry using Raman (**A**) control graphene, (**B**) graphene and PANI and (**C**) graphene, PANI and anti-cortisol.

The morphology of the sensor was assessed during the fabrication process. Raman measurements were taken before and after PANI modification to better understand the structure and defects of the graphene layer (Figure 2) [46]. The control graphene displays three peaks at 1300, 1600 and 2700 cm^−1^, which correspond to the D, G and 2D peaks, respectively [47].

Graphene modified with PANI (Figure 2B) and graphene modified with PANI and Ab (Figure 2C) show a similar spectra compared to the control graphene, with an increased intensity on the D peak. The introduction of a PANI layer on the surface of graphene produces this type of increase on the D peak [48], which is a known Raman spectrum attributed to PANI, indicating that PANI has successfully deposited onto the graphene layer. Other distinct bands attributed to PANI were also visible [47] and overlapping with the standard graphene spectra, but the sp^3^ graphene peak becomes more intense after PANI electropolymerization.

When the anti-cortisol binds, an additional intensification in the peak intensity of the original D and G peaks (Figure 2C) can be observed, showing that anti-cortisol has bound to the PANI graphene layer.

### 3.2. Scanning Electron Microscopy (SEM)

**Figure 3:**
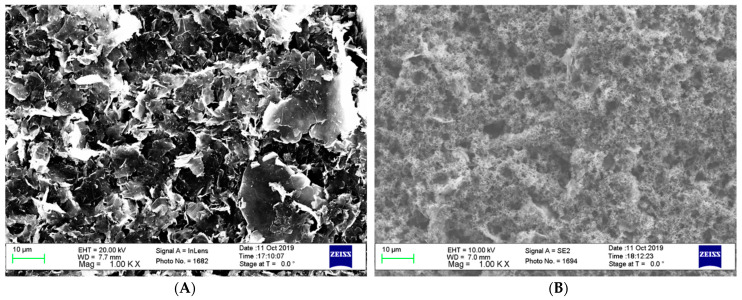
Surface morphology using SEM (**A**) unchanged graphene; (**B**) graphene altered with PANI.

SEM images of the control graphene (Figure 3A) and PANI modified graphene (Figure 3B) surfaces. This imaging allows for visualisation of the surface morphology and further proves that a uniform PANI deposition on the conductive graphene layer was successful. The control graphene shows a smooth surface with some significant topographical features. When PANI is electropolymerized, a rougher surface is introduced, which shows the crystalline form of the polymer [49].

### 3.3. Electrochemical Characterization

**Figure 4:**
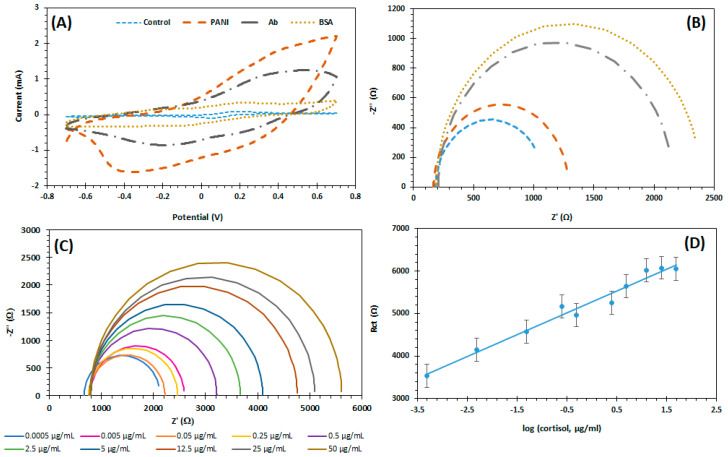
(**A**) CV of control, PANI, antibody and BSA; (**B**) Nyquist plots of control, PANI, anti-cortisol and BSA; (**C**) Nyquist plots for different concentrations of cortisol; (**D**) Calibration curve of R_ct_ values for the different cortisol concentrations.

Figure 4A,B shows the modification layers at the sensor surface. Electrochemical data showed an R_ct_ increased (Figure 4B) compared to control sensor which is observed on the CV data obtained (Figure 4A). The immunosensor shows an increase in the current after the PANI deposition, as seen by [50], and a decrease when Ab and BSA are added onto the surface, because of the conductivity of the same decreases compared to the PANI modified surface, showing a decrease in the potential peak separation (Figure 4A), which is related with the decreased conductivity of the Ab and BSA compared to PANI graphene, resulting in a lower current flow [51].

Figure 4C shows the impedance plots of the fabricated immunosensor with the different cortisol concentrations, and Figure 4D shows the matching calibration curve. The concentration range of cortisol used for the calibration curve was between 0.0005 and 50 µg/mL. On the EIS spectrum, no diffusion-controlled effect was observed [52]. The Rct increased with the increase of cortisol concentration. One of the justifications for this behaviour is that the proteins’ structures when attach to the surface will act as a barrier to the electrical transfer. The immunosensor presents a sensitivity of 0.52 KΩ and a R^2^ of 0.97. The theoretical limit of detection was 3.57 fg/mL.

### 3.4. Controls

Controls were performed to understand the importance of specific binding for the analyte in question. For that, three different processes were conducted, a base graphene, a PANI graphene and an anti-IgG antibody.

**Figure 5:**
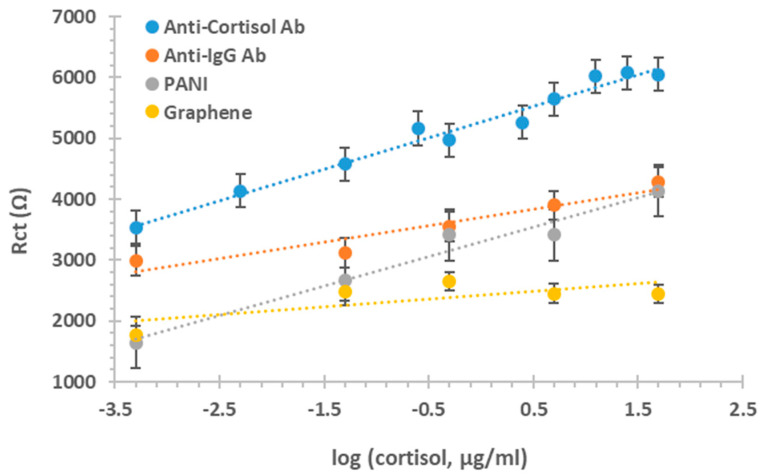
Controls. Calibration curve of Rct values for the different cortisol concentrations in the presence of anti-cortisol antibody (anti cortisol Ab); anti IgG antibody (anti IgG Ab); PANI layer (PANI) and graphene layer (Graphene).

Figure 5 shows that there is a significant difference (*p* < 0.0001) between the anti-cortisol antibody sensor, the base graphene SPE and the PANI graphene SPE detection. The results confirm the affinity of the analyte towards the antibody, and its capability for signal amplification and improved sensitivity [53]. The use of bare graphene-SPE and PANI/graphene-SPEs is not adequate for the detection and quantification of cortisol protein. This is due to the inability of the data to provide an appropriate calibration curve. Between an anti-cortisol antibody sensor and an anti-IgG antibody sensor, a significant difference (*p* < 0.0001) can be seen, confirming the specificity of the cortisol antibody and the capacity to distinguish between different analytes in biological samples. If we assume that the response of each sensor was irreversible because of the bond between antibody antigen interaction, we can access the precision of the sensor comparing values from different sensors. For this, relative impedance was considered and the mean (±standard deviation) of the slope and intercept were 1.6985 (±3.04 × 10^2^) and 0.4379 (±1.34 × 10^2^) for Ab and PANI, respectively. Each sensor is single use, so after calibration, it is not activated anymore, so a direct comparison between them can be done only in relation with the mean relative Rct values (R’, relative to the blank signal of each device). The mean calibration data in graphene and anti-IgG Ab were R’ = 0.1036 × log(cortisol, ng/mL) + 0.9559 (R2 = 0.5238) and R’ = 0.1079 × log(cortisol, ng/mL) + 0.4936 (R2 = 0.9083), respectively. The calibration performed with anti-cortisol Ab is the greatest in terms of analytical performance.

### 3.5. Interference of the Immunosensor

**Figure 6:**
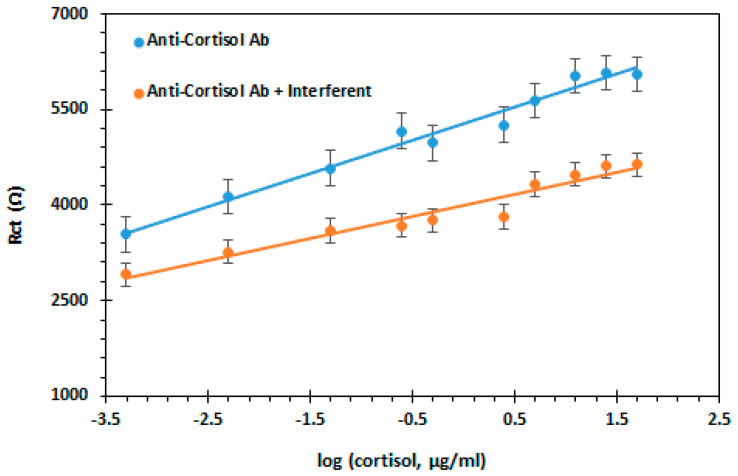
Calibration curve of cortisol and of progesterone interference.

Selectivity was performed to evaluate the immunosensor to cortisol and understand non-specific binding; a different hormone, progesterone was used. The calibration curve results are shown in Figure 6. A linear reply from 0.0005 to 50 µg/mL, with a slope of 1.1 kΩ/[cortisol, µg/mL] were detected. Comparing both the cortisol 1.1 kΩ/[cortisol, µg/mL], and progesterone 0.64 kΩ/[progesterone, µg/mL], we can observe a decrease in sensitivity when using the interfering, suggesting that the immunosensor has good selectivity.

### 3.6. Evaluation of Cortisol Levels in Canine Saliva Sample

Values of cortisol in saliva samples from dogs were determined using impedance values on calibration of BSA/anti-cortisol/PANI/graphene-SPE with saliva samples collected from guide dogs. The samples were collected on two different days, before and after training, as shown in Table 2. The samples tested show an increase of approximately two-fold in salivary cortisol concentrations after the training session. However, one dog (Flora) was the only one that appears to have had considerably higher cortisol levels, and indeed this dog was a very stressed pup, according to the trainer, which makes this result accurate with the condition of the dog. All other samples were not meaningfully above or below the average cortisol level, suggesting little to no stress in the dogs. Cobb et al. [3], documented a cortisol concentration range of 0 to 337.9 ng/mL, with a mean of 4.5 ng/mL and a median of 1.5 ng/mL on studying meta-analysis of salivary cortisol levels.

## 4. Conclusions

In this study, we present a simple modification approach to a graphene immunosensor for antibody binding with a suitable orientation for antigen binding. The resistance of the immunosensor increases with the increase of log(cortisol) concentration. The sensors demonstrated a good analytical performance in addition to measuring a cortisol concentrations applicable to the physiological levels present in dog saliva with a linear detection range from 0.0005 to 50 μg/mL and a theoretical limit of detection of 3.57 fg/mL. This immunosensor can be used as a POC device to assess welfare in dogs.

## Figures and Tables

**Table 1:** Sensors for cortisol using different detection techniques.

Electrode Support	Immunosensor Design	Detection Technique	Response to Cortisol	Antibody Binding	Ref.
—	Cortisol in saliva and plasma was measured by use of a competitive electrochemiluminescence immunoassay (ECLIA).The required sample volume was 300 mL saliva or plasma	ECLIA	The ROCHE Elecsys immunoassay analyzer correctly measured salivary cortisol in dogs. However, a broad clinical application of the method seems limited, because of the large sample volume required.	—	14
NiO/ITO	NiO/ITO was hydroxylated, after a silanization with APTES, followed by a EDAC/NHS chemistry for anti-cortisol binding.	CV and DPV	LR: 1 pg/mL–10 μg/mL LOD: 0.32 pg/mL	Via -NH_2_ groups	22
IDEs	Anti-Cab covalently immobilized on SAM of DTSP modified IDEs. The non-binding sites of immunosensor surface were blocked using EA.	CV	LR: 10 pg/mL–100 ng/mL LOD: 10 pg/mL	Via -NH_2_ groups	23
AuNPs/MrGO	Electrode was modified by AuNPs. Cor can be firmly loaded in the AuNPs/MrGO. Finally, the detection signal of electrochemical immunosensor (HRP-Strept-Biotin-Ab Cor/AuNPs/MrGO/Nafion@GCE)	CV, DPV and EIS	LR: 0.1–1000 ng/mL LOD: 0.05 ng/mL	HRP-Strept-Biotin-Ab	24
Graphite SPEs	AP conjugate synthesized with disposable graphite SPEs. 1-NP was used as an enzymatic substrate.	SWV	LR: 0.2–44.6 ng/mL LOD: 0.6 ng/mL	blocked with PVA	9
Paper-based	Fabricated using a simple and cheap wax printing method. Cortisol conjugated-BSA was immobilized on the paper’s surface in the detection zone for the competitive immunoassay. Anti-cortisol mAb-conjugated gold nanoparticles, as signal indicator, were used to detect cortisol in the sample.	Colorimetric	LR: <0.25 μg/mL, 0.25–0.5 μg/mL and >0.5 μg/mL LOD: 0.215 μg/mL	—	25
Au electrode	The cortisol detection relies on a competitive immunoassay format using the ALP enzymatic tag along with 1-naphthyl phosphate (1-NPP) enzymatic substrate.	Chronoamperometry	LR: 0–250 ng/mL LOD: 13.4 ng/mL	Via -NH2 groups	26
ITO	ITO electrode surface was done in two steps; deposition of nitrobenzene group to yield nitrobenzene-modified ITO and nitrobenzene reduction to aniline. Subsequently, Cortisol antibodiy was chemically labelled with a redox tag (ferrocene).	CV and SWV	LR: 0–50 ng/mL LOD: 1.03 pg/mL	Via -NH_2_ group	27
Screen printed carbon electrodes	Bare electrode was modified by fullerenes, then an in-situ polymerization was performed by using APS to start acrylamide polymerization via the carboxylated surface and to form polymers around cortisol.	CV and EIS	LR: 0.18 μg/mL and 23.2 μg/mL LOD: 0.05 μg/mL	Via -COOH groups	28
ITO	ITO electrodes were pretreated with a 5:1:1 solution of H_2_O and NH_4_OH. ITO-cortisol-BSA was then added to the surface and next anti cortisol IgG−DI conjugate was immobilized onto the modified electrodes.	CV and Chronocoulograms	LR: 0.011 μg/mL–0.445 ng/mL LOD: ∼0.011 μg/mL	Via -NH_2_ groups	29
IDE-Au	Anti-Cab was covalently immobilized onto a DTSP-SAM/IDE-Au electrode. Anti-Cab/DTSP-SAM/IDE-Au bioelectrodes were blocked with EA.	CV	LR: 10 pg/mL–500 ng/mL LOD: 10 pg/mL	Via -NH_2_ groups	30
Glassy carbon	GCEs were first polished and then drops of a mixture of 5 M SnS2 were deposited on the surface of pretreated GCEs. Solutions of anti-cortisol antibody and BSA were prepared in PBS.	CV, EIS and DPV	LR: 44.5 ng/mL–36,246 μg/mL LOD: 44.5 ng/mL	—	31
SPEs	SPEs were activated with NaOH. Next, the first layer of AuNPs is added Then MoS2 was dropped evenly onto the working to form stable Au–S, and an effective SAM of MoS2 was created.	DPV	LR: 0.18–72.5 μg/mL LOD: 0.039 μg/mL	—	32
AuNPs	Four different colorimetric cortisol analyses that use various chromogens, which include sulfuric acid, Porter−Silber reagent, Prussian blue, and blue tetrazolium, are studied.	Colorimetric	LR: 0.05–2 μg/mL LOD: 97 ng/mL		33
Au chip	The surface of an Au chip was modified with PEG6-COOH aromatic dialkanethiol self-assembled monolayers and hydrocortisone 3-(O-carboxymethyl) oxime (hydrocortisone 3-CMO) as a cortisol analog.	SPR detection	LR: 1 × 10^−8^ µg/mL–0.1 µg/mL LOD: 3.8 × 10^−8^ µg/mL	—	34
—	Samples submitted for testing were incubated with a biotinylated antibody and with a cortisol derivate marked with ruthenium. Micro particles coated with streptavidin were added, and by the magnetic effect, formed complex was attached to the solid phase. Next, the unfixed forms were separated, and the electric voltage was applied to induce chemiluminescence.	ECLIA	—	—	35
—	One hundred μL of serum sample was directly extracted by adding 2 mL ethyl acetate, followed by chromatographic separation on a C18 column with a mobile phase consisting of 5 mM ammonium acetate and methanol.	LC-MS/MS	LR: 1.0–500.0 ng/mL LOD: 0.2 ng/mL	—	36
Platinum/graphene	Platinum/graphene aptamer extended gate field effect transistor (EG-FET) for the recognition of cortisol.	EG-FET	LR: 0.36 µg/mL and 3.62 µg/mL LOD: 0.07 µg/mL	—	37
Paper substrate	Inkjet printed on a paper substrate with a metalloporphyrin based macrocyclic catalyst ink that can electrochemically reduce cortisol, captured by aptamer functionalized magnetic nanoparticles.	EIS	LR: 0.036 µg/mL–18.1 µg/mL LOD: 0.0036 µg/mL	—	38
Au nanoparticles	A duplex aptamer conjugated to the surface of Au nanoparticles (AuNPs) by Au–S bonds is utilized as the sensor probe in a LFA device.	LFA	LR: ∼0.5–15 ng/mL LOD: 0.37 ng/mL	—	39
Au nanoparticles	Lateral flow assay for cortisol detection using aptamer-conjugated AuNPs	LFA	LR: ∼10–100 ng/mL LOD: ∼1 ng/mL	—	40
Screen-printed graphene electrodes	The cortisol DNA aptamer was modified with streptavidin magnetic beads (MBs) before simple immobilization onto the electrode surface using a neodymium magnet.	EIS and CV	LR: 0.10–100 ng/mL LOD: 2.1 pg/mL	—	41
Polyamide	Aptamer molecule was functionalized on ZnO coated nano-porous polyamide substrate-based sensor.	EIS and CA	LR: 1–256 ng/mL LOD: ~1 ng/mL	—	42
Silver nanoclusters	The aptamer structure has sequence against cortisol in the middle and two cytosine reach compartments in 3’ and 5’ ends which are designed for Ag-NC supramolecule configuration. The molecule-like properties of the Ag nanocluster create a strong AIE fluorescence signal. Binding aptamer to cortisol causes proximity of Ag-NCs supraparticles and leads to aggregation base fluorescence enhancement.	AIE fluorescence signal.	LR: 0.36 µg/L–326 µg/L LOD: 0.36 µg/L	—	43
Quantum dots	Anticortisol antibodies or cortisolselective aptamers tethered on CdSe/ZnS core−shell QDs. The aptamer-conjugated or antibody-conjugated QDs were carried by ∼20 nm-sized magnetic nanoparticles (MNPs) to form aptamers−QD@MNP or antibody−QD@MNP nanosensors,	Fluorescence quenching	LR: 0.145–145 µg/mL LOD: ∼0.362 µg/mL	—	44

PBS: Phosphate-buffered saline; DPV: Differential pulse voltammetry; SWV: Square-wave voltammetry; CA: Chronoamperometric; CV: Cyclic voltammetry; GCE: glassy carbon electrode; APTES: 3-aminopropyltrimethoxysilane; HRP: horseradish peroxidase; EG-FET: extended gate field effect transistor; EIS: Electrochemical Impedance Spectroscopy; ITO: Indium Tin Oxide; EDAC: N-(3-dimethylaminopropyl)-N’-ethylcarbodiimide; NHS: N-hydroxysuccinimide; AP: alkaline phosphatase; SAM: self-assembled monolayer; DTSP: dithiobis(succinimidylpropionte; IDE: interdigitated microelectrodes; EA: ethyleneamine; AuNPs: gold nanoparticles; MrGO: magnetic functionalized reduced graphene oxide; Cor: cortisol; SPEs: screen printed electrodes; 1-NP: 1-nalphtyl phosphate; NiO: Nickel Oxide; PVA: polyvinyl alcohol; ALP: alkaline phosphatase; SPR: Surface plasmon resonance; LFA: lateral flow assay; ECLIA: Electro-chemiluminescence immunoassay LC-MS/MS: liquid chromatography-tandem mass spectrometry BSA: PEG6-COOH: aromatic dialkanethiol. AIE: Aggregation Induced emission.

**Table 2:** Cortisol levels in IGDB saliva samples measured by the immunosensor.

Dog	[Cortisol] (ng/mL)-Day 1		[Cortisol] (ng/mL)-Day 2	
	Before	After	Before	After
1	45.9 ± 0.21	194.0 ± 0.69	2.81 ± 0.04	5.95 ± 0.58
2	1.25 ± 0.13	3.61 ± 1.46	0.70 ± 0.36	2.22 ± 0.50
3	0.39 ± 0.01	0.79 ± 0.08	1.24 ± 0.07	1.16 ± 0.23
4	-	-	3.49 ± 0.32	7.49 ± 0.59

## Data Availability

Not applicable.
